# Who Has Higher Willingness to Pay for Occupational Safety and Health?—Views from Groups with Different Public Identities and Differences in Attention

**DOI:** 10.3390/ijerph15081667

**Published:** 2018-08-06

**Authors:** Shanshan Li, Hong Chen, Xinru Huang, Ruyin Long

**Affiliations:** School of Management, China University of Mining and Technology, Xuzhou 221116, China; shanshanli0809@163.com (S.L.); hxr881003@163.com (X.H.); longruyin@cumt.edu.cn (R.L.)

**Keywords:** occupational safety and health, attention, identity discrepancy, WTP

## Abstract

Background: Occupational safety and health issues are closely associated with the wellbeing and survival of every worker and family, as well as of society as a whole. It is a type of typical public issue and requires cooperative governance among different governing subjects. Methods: According to the questionnaire investigation on 2179 subjects with different identities, the research explored the willingness to pay (WTP) for occupational safety and health and the degree of attention, with different identities, through the difference analysis and descriptive statistical analysis. The research studied the relationship between public attention and WTP through the methods of cross-analysis, correlation analysis, and regression analysis. Results: (1) The public show a disregard attitude to occupational safety and health. (2) The public expect the government to fund and solve occupational safety and health problems rather than for themselves to pay directly. (3) Over 50% of questionnaire respondents defined occupational safety and health problems as being classified into two categories, namely, “no attention—government payment” or “no attention—refusal of individual payment”, according to the analysis. (4) The level of attention paid to occupational safety and health can significantly predict the individual income WTP, item WTP, subject WTP, and event WTP. Conclusions: This research aimed to outline the implications for the governance of occupational safety and health.

## 1. Introduction

Occupational safety and health appeared along with the industrialization and urbanization process in Europe and the United States, and attracted much attention in all countries during the early 20th century. Subsequently, a lot of research has been carried out [1,2]. Occupational safety and health involve ensuring the safety and health of laborers, and social economic development is the factor that maintains the functional entirety and benign circulation of the production system. However, the current state of occupational safety and health measures is not adequate. According to the preliminary estimation issued by the International Labor Organization, every 15 s, about 160 workers experience occupational accidents throughout the world, and one worker will die due to occupational accidents or diseases. Moreover, there are around 160 million non-lethal occupational disease cases each year, and the losses due to occupational diseases and accidents each year total up to 1.25 trillion dollars [3]. The occupational safety and health conditions in China are becoming increasingly severe. According to data from the Ministry of Public Health, over 16 million companies were engaged in poisonous and hazardous operations in 2011, and 200 million workers were affected by occupational diseases to varying degrees in the labor process [4]. The annual state economic losses reported by industrial injury accidents and occupational diseases in China have reached to more than 200 billion RenMinBi (RMB) and account for 2.5% in gross domestic product (GDP) terms [4]. Occupational hazards and occupational diseases have become the prime factors for influencing the physical health of laborers, and cause premature loss of laborers’ working capacities.

Safe and healthy production are two perpetual propositions for the survival and existence of human beings. Occupational safety and health problems have become a common concern of the government, third-party social subjects, diverse employing units, and laborers. Different subjects have different positions in the society. As a result of this, specific rights, obligations, responsibilities, loyal subjects, identities, and acting rules could find legitimate causes. The “identity” proposed in the social identity theory emphasizes the relationship between groups and groups, where it assumes that the groups to which individuals belong (such as political groups, clubs, and nationalities) can give a precise definition of identity for the group members [5]. Guo considered the major effects of the identity concept on human life, work, and other aspects under the traditional concepts in China, by analyzing the value orientation of the identity system and probing the rich awareness of the citizens, thus deriving the conclusion that Chinese people in modern society still have a strong identity complex and concept [6]. The public attitude and behavior towards occupational safety and health based on identity differences are bound to be different. For example, front-line mine staff, as a high-risk group facing occupational safety and health problems, lack the relevant knowledge and awareness, which would possibly cause frequent health risk behaviors. The coal mine corporate leaders represent the will of enterprises, and they face dual-constraints by national policies and regulations, and economic benefits. Previous research focused on occupational safety and health from a single perspective, such as the perspective of coal workers [7], coal mining enterprises [8], or the government [9]. However, the perceptive of identity difference was ignored in the research of occupational safety and health. Based on the difference of subject identity, this study offers a new perspective and a new route to the occupational safety and health management practices in China.

There is a growing trend in re-orientating occupational safety and health research; such a trend is accelerated by the increasing attention to occupational safety and health. As a starting point for public participation, public attention refers to the attention to certain thing by social groups. Public attention is an important indicator for measuring the strengths and weaknesses of the internal relationships between objects and group behaviors, where it can have major effects on the orientation of public opinion, culture, and policies [10]. Spijkers and Honniball suggested that public participation contributes to the handling of public services where public attention is the starting point of public participation [11]. Individual attention to public and social services has very important effects on attitudes and behaviors during public participation processes. The attention that studying is given to public occupational safety and health could help to supervise and control the present conditions of occupational safety and health. Willingness to pay (WTP) refers to the willingness of consumers to pay for products or services [12]. Many studies have investigated the WTP among consumers [13,14,15,16]. However, studies of the WTP for public issues have focused mostly on environmental pollution [17,18], renewable resources [19,20], and water resource protection [21,22], whereas few studies have considered the WTP for occupational safety and health. From an economic perspective, the governance of occupational safety and health does not have a market price as a public service. Studying the WTP by different subjects for occupational safety and health governance can help identify the market preferences for occupational safety and health, thereby providing policy evidence for the government’s governance of occupational safety and health problems.

Attention could have a significant impact on the individual mode of behavior. WTP is a form of individual behavior. Scholars have carried out a lot of empirical research on the relationship between attention and behavior. For example, in the field of the environment, Vringer et al. believed that attention to environmental issues significantly influenced residents’ energy consumption behaviors [23]. Groot and Steg argued that environmental cognition, environmental knowledge, and the degree of attention to the environment significantly influence the residents’ choice of travel modes [24]. In the field of finance, Seasholes and Wu believed that individual investors’ attention to stocks could affect their amount of investment [25]. Shao took emergencies as examples to study the rhetoric strategy and communication path of news release from the perspective of public concern [26]. This study speculates that there would exist a relationship between public concern for occupational safety and health and WTP.

**Figure 1:**
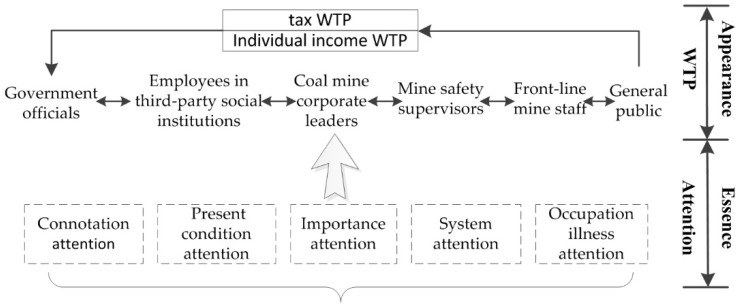
The roadmap of the relationship between the attention to occupational safety and health and willingness to pay (WTP) based on different subject identities.

By 2016, the number of people employed in various industries in China had reached 776 million [27]. Occupational safety and health problems involve every family. However, the solution of occupational safety and health problems depend not only on the relevant technology and the appropriate management mode, but also on the public attitude, concern, and support for occupational safety problems. Therefore, studying occupational safety and health based on differences in identity may be helpful for clarifying the basis of governance for occupational safety and health. Few scholars have studied the relationship between public attention and WTP. This study explores the relationship between the attention to occupational safety and health and their WTP based on subject identity differences, which has important theoretical and practical significance in promoting occupational safety and health governance in China. The roadmap of the relationship between the attention to occupational safety and health and WTP based on different subject identities (Figure 1).

## 2. Literature Review

This study considers that there is a certain correlation between public attention and public cognition, and both of them are positive public participation processes. Li and Liu regarded public knowledge cognition as the prime focus of public participation cognition [28]. Li illustrated urban residents as the research objects, and holds that public participation cognition contains the degrees of knowledge and cognition for public affairs and social affairs [29]. Therefore, in the present study of attention to occupational safety and health attention, there is an emphasis on the public awareness of relevant occupational safety and health knowledge and the public cognition of occupational safety and health. This study defined attention to occupational safety and health as referring to caring about occupational safety and health attention problems and being familiar with the present conditions and various relevant knowledge.

In recent years, scholars have gradually paid closer attention to the WTP of public affairs. For instance, Sun et al. take the Beijing Plain Greening Project as the background to point out that 57.8% of the subjects voluntarily contribute to environmental construction [30]. Soon and Ahmad carried out a study on water resource WTP and find that more and more families have a WTP for water resource protection [31]. Occupational safety and health is a typical public issue. First, safety and health are essential requirements. Occupational safety and health are closely related to the survival and development of every laborer. Consequently, every laborer and their family will benefit from improvements to the occupational safety and health guarantee level. Second, safety and health have non-competitiveness consumption features. Any laborer has access to the enjoyment of the quality environments created by improvements to occupational safety and health, and other laborers should not be prevented from enjoying the same benefits. Third, safety and health have non-exclusive profitability features. Even if laborers do not want to pay fees to ensure their occupational safety and health governance, they should not exclude this option if they are in the income range required to improve their occupational safety and health level. In other words, every laborer would benefit from it. Therefore, scholars’ studies on the WTP for public affairs would positively support this study. 

The main methods of measuring the WTP include the contingent valuation method (CVM), experimental auctions, conjoint analysis, and choice experiments [32]. Due to improvements in the CVM theory and the methods employed, there have been many advances in determining the WTP, analyzing the possible factors involved, and statistical analysis methods. CVM overcame limitations in the absence of an actual market and alternative market exchange products so that it can be used as a unique method for evaluating the overall usage values and non-usage values related to public issue. The simulated market method has been applied widely in various fields, including studies of the ecological environment [33], public sanitation [34], food consumption [35], and lifestyle and health [36].

## 3. Methods 

### 3.1. Measuring Variables

#### 3.1.1. Measuring of Public Attention on Occupational Safety and Health

Given the lack of previous studies on public attention to occupational safety and health public attention, this study conducts an explorative and qualitative study to collect materials through interviews with 43 residents from different industries, ages, and identity features. The interview outline concretely included the following items; for example, “Do you have an understanding of issues related to occupational safety and health?”, “Could you provide additional details about your knowledge of these issues?”, “Have you paid any particular attention to the issue of Occupational safety and health?”, and “Could you tell us what aspects of occupational safety and health you have focused on?”.

This study conducted a text analysis with a grounded theory based on the process of open coding and axial coding as well as selective coding [37,38]. The researchers used computers to analyze qualitative data software (CAQDAS) to categorize and segment qualitative data that comprised a large amount of text, to facilitate archiving and lookups, as well as the marking of text fragments with Indep as a code. In summary, the 94 entries examining the attention paid to occupational safety and health were collected, and were further coded and labeled. The 12 entries that consisted of ambiguous items were deleted. Then, the remaining 82 entries were classified such that entries that were deemed the same or extremely similar were allocated to one category. Thirty-six original concepts on occupational safety and health were obtained following the process of conceptualizing the raw text data collected from questionnaire responses. For example, such themes included “The status of occupational safety and health is not optimistic”, “The number of occupational diseases is the highest in the world”, “Occupational safety and health lags behind economic and social development”, “Public health is placed in the strategic position of China”, “Approximately 80% of occupational-related illnesses can be attributed to pneumoconiosis”, and so on. Due to the complexity of the 36 original concepts, the researchers invited eight professionals (i.e., two professors, two associate professors, and four lecturers) to work together to simplify the 36 concepts. Following discussion, sorting, and concise classification, the final 13 items on the scale were agreed upon. In the end, the above items were divided into five dimensions comprising connotation attention, present condition attention, importance attention, system attention, and occupation illness attention.

This paper used a self-reporting method to measure the level of attention paid to Occupational safety and health. A five-point Likert scale was used for the responses, where 1 = “do not agree at all”, 2 = “do not agree to some extent”, 3 = “unsure”, 4 = “agree to some extent”, and 5 = “totally agree”. Higher scores indicated greater attention to occupational safety and health. The subjects completed the assessment according to their actual attention levels. The example items are shown in Table 1 (See the Appendix A for all items).

#### 3.1.2. Measuring WTP

At present, the government is guider of the governance for occupational safety and health when solving relevant occupational safety and health social problems, the expenditure of which is funded by part of financial costs sourced from the public payment of taxes. Therefore, studying whether the public are WTP for a tax to support occupational safety and health could help to understand the willingness of the public and to financially improve to the governance of occupational safety and health problems. In addition, the development of occupational safety and health systems requires public participation. Determining the direct WTP for occupational safety and health measures could facilitate the improved governance of occupational safety and health problems. Direct public support for occupational safety and health measures might comprise the following. The public could donate some of their earnings to fund occupational safety and health governance “measures”, such as setting up a special occupational safety and health fund, and insurance for occupational disease patients. In addition, the public could directly fund occupational safety and health “events” or “subjects” such as designated donations for major occupational safety and health accidents, accident victims, and occupational disease patients.

Thus, the measures employed to measure the WTP for occupational safety and health in this study mainly comprised measurements of the tax WTP for occupational safety and health (taxpayer perspective) and measurements of the individual income WTP. The individual income WTP included the item WTP (individual willingness to support the country in solving occupational safety and health problems with personal earnings), event WTP (individual willingness to directly donate to help major occupational safety and health accidents), and subject WTP (individual willingness to directly donate to accident victims or occupational disease patients with personal earning).

### 3.2. Contingent Valuation Method

We used CVM to evaluate the degree of support for occupational safety and health measures. The principle was to utilize the maximum utility theory and adopt a questionnaire to reveal the consumers’ preference and WTP for a certain public article through offering consumers a hypothetical market, thus further assessing the economic values of such a public article [39].

In the CVM assessment, open-ended (OE) questionnaires and payment cards (PC) were chosen as the research methods in this study. The description of a scenario is the most important component of CVM [40]. The specific scenario in the questionnaire was as follows.

“The financial expenditure budget by the central government for public safety (stability maintenance and emergencies) in 2017 is 183.855 billion RMB and the corresponding per capita budget is 133.71 RMB. If the country added occupational safety and health to the agenda of the financial expenditure budget, should the per capita public safety level be increased or reduced in your opinion?”, “If you were required to help the country to solve occupational safety and health problems with your personal income (such as setting up special occupational safety and health funds and purchasing insurance for occupational disease patients), what percentage of your income would you be willing to pay?”. The study design incorporated the per capita per month form as the payment unit for the bidding sum so as to more authentically reveal the WTP of the subjects.

In order to determine an appropriate payment card bidding starting point and value interval for the CVM, it was first necessary to conduct a basic preliminary investigation. We collected 200 valid questionnaires with an effective response rate of 100%. Among the 200 questionnaires, 162 subjects had positive WTP (81%) and 38 subjects had zero WTP (19%). Table 2 illustrates the distribution of the WTP in terms of the bidding amounts and the WTP responses as the bidding sum under the condition that WTP > 0.

As shown in Table 1, the WTP bidding amounts by the subjects were mainly 0%, 1%, 3%, and 10% (per capita per month), where 95.5% of the subjects had a WTP of 10% per capita per month or below. Most subjects had a WTP that was 1% of their income (per capita per month) and the maximum was 26.5%. Thus, we determined the payment card bidding starting points and interval values as: 0%, below 1% (excluding 0% and 1%), 1–3% (excluding 3%), 3–10% (excluding 10%), and above 10% of the individual’s income.

Since subjects with identity discrepancies have different statuses in the society, they also have different value orientations and judgment stances on things. Therefore, this study designed specific questionnaires for the different research subjects, including the basic information of the different identity subjects, their attention to occupational safety and health, and their WTP for occupational safety and health. The basic information obtained included differences in identity, gender, age, marital conditions, monthly salary, residence area, educational background, and other social variables. The attention scale for occupational safety and health included 13 items. Similarly, the WTP scale for occupational safety and health contained four items.

### 3.3. Preliminary Investigation

The preliminary investigation was conducted in March 2017 with the six aforementioned subject groups. We distributed 500 questionnaires and collected 433 valid questionnaires. Descriptive statistics for the preliminary investigation samples showed that the gender proportion was reasonably well balanced (males = 53.6% and females = 46.4%), and the age groups were well distributed (<25 years = 10.4%, 26–30 years = 21.5%, 31–35 years = 14.7%, 36–40 years = 7.3%, 41–45 years = 18.6%, 46–50 years = 11.1%, and >50 years = 6.4%). Having tested the validity and credibility of the questions, the reliability and validity of the questionnaire were analyzed by the method of items analysis and principal component analysis, which proved the scale had good reliability and validity. The formal preliminary investigative questionnaire underwent revision.

### 3.4. Ethical Approval

This study was carried out in accordance with the recommendations of the Ethical Codes of Consulting and Clinical Psychology of Chinese Psychological Society, Chinese Psychological Society. The protocol was approved by the China Occupational Safety and Health Association—Occupational Mental Health Professional Committee. All subjects gave written informed consent in accordance with the Declaration of Helsinki.

It is the duty of researchers who are involved in psychological research to protect the life, health, dignity, integrity, right to self-determination, privacy, and confidentiality of personal information of the research subjects. The responsibility for the protection of research subjects always rested with our research team and the China Occupational Safety and Health Association—Occupational Mental Health Professional Committee, and never with the research subjects, even though they had given consent.

## 4. Research

### 4.1. Subjects

We selected front-line mine staff, coal mine corporate leaders, mine safety supervisors, government officials, employees in third-party social institutions, and the general public as the research subjects. The rationale behind this decision may be understood in terms of the following.

Pneumoconiosis is the most common occupational disease in China. Pneumoconiosis patients account for ~80% of all the patients afflicted by occupational diseases, and the coal industry accounts for >60% of the total pneumoconiosis cases reported. Therefore, the coal industry offers a solid theoretical and practical basis for a study examining occupational diseases. At present, China has 6000 coal companies, including 45 large and medium-sized coal companies. The proportion of large-scale coal bases has reached 93%. Given this situation, we selected three representative companies from the 45 large and medium-scale state-owned coal companies for this investigation. In addition, to ensure the representativeness of the sample distribution, we selected mine staff and leaders from six different positions related to ventilation and fire prevention, coal mining, tunneling, mechatronics, transportation, and ground work, as well as mine safety supervisors, according to their different ages and positions.

In parallel to the growing complexity of public affairs, cooperative governance has become the primary mainstream international modal for the governance of public affairs, and it is dependent on the participation of numerous social institutions. Among these, the role of third-party social institutions (i.e., including various occupational disease hospitals, occupational disease relief foundations, and industry association institutions) should not be overlooked. China has 72 occupational disease hospitals that cover each provincial administration unit. We randomly selected three hospitals with different sizes in several provinces among the 72 occupational disease hospitals, and we surveyed workers in these hospitals who had different positions and ages. The Chinese Occupational Safety and Health Association (COSHA) was established in 1983 as an organized institution with a council consisting of health and safety experts across all industries. In representing a high proportion of workers and institutions, COSHA has become one of the most powerful associations in the field of occupational safety and health in China. The China Coal Mine Pneumoconiosis Prevention Foundation was established in 2003 and it is now a nationwide public welfare charitable organization. With 17 volunteer teams, it has treated approximately 160,000 mine staff affected by pneumoconiosis. In addition, some small organizations have grown rapidly, such as Love Save Pneumoconiosis which was established in 2011 and aims to provide assistance to the six million farmers in China who suffer from pneumoconiosis. In July 2017, Love Save Pneumoconiosis had >8000 volunteers across 26 regions. The researchers in this study selected employees and volunteers from COSHA, the China Coal Mine Pneumoconiosis Prevention Foundation, and Love Save Pneumoconiosis in terms of their age, educational background, and position.

In China, the government acts as the absolute subject in occupational safety and health governance and takes charge of relevant capital investments, policy enactments, measures of implementation, and accident rehabilitation. In this sense, it is necessary to investigate the government’s official attention and WTP. At the same time, the development of occupational safety and health governance is inseparable from the common social public because their attention and support directly decide the social atmosphere that is confronted by occupational safety and health problems. The government officials and members of the general public were selected using the group sampling method. According to the different economic and regional features in the eastern, middle, and western areas of China, we selected two cities from each of the three areas (Hebei and Jiangsu Province in the east, Anhui and Hunan Province in the middle, and Sichuan and Xinjiang Province in the west). The subjects included employees who differed in terms of their gender, educational level, marital status, and occupations.

### 4.2. Samples

The formal investigation was conducted during April–July 2017. Considering the wide distribution and large quantity of features collected in the samples, eight members participated in the investigation by collecting data with study questionnaires. The questionnaires were distributed by means of the paper questionnaire and network questionnaire.

The paper questionnaire was used for respondents comprising front-line mine staff, coal mine corporate leaders, and mine safety supervisors. In addition, in view of the poor cooperation of coal miners in investigation, more than 20 large and medium-sized state-owned coal mining enterprises, which had cooperated with our research group, were selected. Following communication with the enterprise leader about the aims of the study, the leader’s consent was obtained. The survey was then formally conducted either before or after the shift meeting to avoid causing any hindrances to their formal work. Notably, the educational level of the front-line mine staff was low as a whole. As such, the questionnaires were completed by the survey team with centralized guidance provided to each group. Members of the survey team liaised with no more than five miners to ensure the authenticity and integrity of information throughout the data collection process. Prior to filling out the questionnaire, the specific members of the survey team provided a unified explanation and the main outline of the questionnaire to the subjects and told them the issues that required attention. With respect to the employees in third-party social institutions, the network questionnaires were conducted via WeChat. Internal WeChat groups of members from the organizations including the Occupational Safety and Health Association, the China Coal Mine Pneumoconiosis Prevention Foundation, and Love Save Pneumoconiosis exist. The researchers then obtained the permission of WeChat group managers to distribute the questionnaires to all WeChat groups. To ensure the overall quality of questionnaires, only those questionnaires that were completed in >5 min were deemed to be valid, due to the number of items listed. It was worth noting that the WeChat survey was also anonymous. Paper questionnaires were used with respect to government officials and the general public, members of whom were authorized, and comprised other students, friends, and relatives of the survey team, etc. Members of the survey team selected some government officials at the government gate in order to negate the shortfall in the sample.

Notably, the investigators informed participants about the purpose of the study, stating that such information would be used for the sole purpose of scientific application. Prior to each investigation, the researchers gave assurances to participants that the individual information collected was confidential. During this time, researchers placed emphasis on the importance of providing truthful answers to questionnaires. Finally, the researchers thanked respondents for their participation in the investigation by offering small gifts or WeChat lucky money, which further increased the response rate and effectiveness of the questionnaire. We distributed 2500 formal questionnaires and collected 2197 valid questionnaires with a response rate of 87.16%. Ethical approval is same as “Section 3.4”. The structure of the samples is listed as Table 3.

### 4.3. Reliability and Validity Test

According to reliability and validity tests based on the formal questionnaires, the overall Cronbach’s α value for attention to occupational safety and health was 0.917, which indicated that the scale generally had higher reliability. The Cronbach’s α values for all the potential variables ranged between 0.835 and 0.922, and the CR values ranged between 0.803 and 0.911, thereby conforming to acceptable standards and passing the test of reliability.

**Figure 2:**
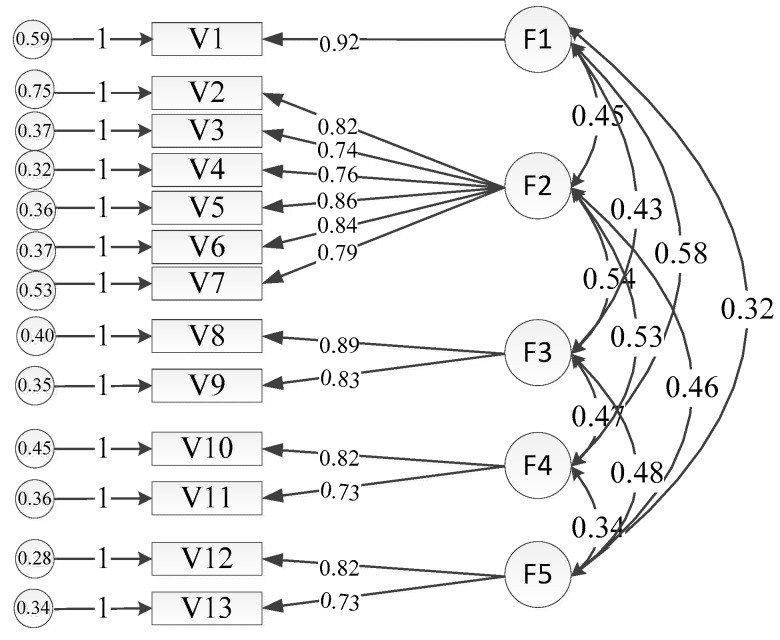
Estimations of the standardized path coefficient of the confirmatory factor model.

The development of the attention to occupational safety and health scale strictly followed a scale development program where valid content was determined after discussions and revisions by experts. The AMOS 17.0 method was used for the confirmatory factor analysis. The standardization method is provided in Figure 2. The statistical analysis showed that the goodness of fit test parameters for the model (χ^2^/df = 5.790, RMSEA = 0.047, GFI = 0.972, IFI = 0.973, CFI = 0.973, TLI = 0.964) were all within the acceptable ranges, and thus the scale had relatively favorable structural validity. The AVE value for factors between 0.559 and 0.716 satisfied the condition of AVE > 0.5, which showed that the scale had favorable convergent validity. In addition, the AVE square root of the potential variables was above the correlation coefficient among the potential variables, and the variable potential structure had a relatively good discrimination index, and so the scale passed the test of validity.

## 5. Data Analysis and Results

### 5.1. Difference Analysis

This study adopted independent sample statistical analyses and one-way ANOVA to analyze the discrepancies of public occupational safety and health attention and dimension, tax WTP, individual income WTP, item WTP, event WTP, and subject WTP in the context of the demographic variables and organization work variables. The specific analysis results are shown in Table 4.

As shown in Table 4, there were significant differences in the attention to public occupational safety and health, tax WTP, individual income WTP, item WTP, event WTP, and subject WTP, according to identity and age. There were significant differences between the attention to occupational safety and health, and system attention according to gender. There were significant differences among connotation attention, present condition attention, individual income WTP, item WTP, and event WTP according to marital status. There were significant differences among the attention to occupational safety and health attention and dimensions and the individual income WTP and dimensions in the monthly income and education level aspect. There was also a significant discrepancy between the occupational safety and health attention and dimensions in the aspect of the residence area. We specifically selected the differences in variables related to identity, to investigate the attention to occupational safety and health.

### 5.2. Descriptive Statistical Analysis

Table 5 shows that the overall mean value of the attention to occupational safety and health by different subjects was below 3, where about 80% subjects gave little or no attention to occupational safety and health attention problems. Thus, the general public usually gave little consideration to occupational safety and health. For attention to occupational diseases, the corresponding mean value was 2.710. In agreement with the WTP analysis, the public tax WTP for occupational safety and health (M = 3.746) was far higher than the individual income WTP for attention to occupational safety and health (M = 2.529), and item WTP < subject WTP < event WTP. Only 13.77% of the subjects had negative attitudes regarding their WTP for an occupational safety and health attention tax, which suggests that the public accepted and supported central government-funded occupational safety and health governance, but that the degree of support still required further improvement. Overall, 66.36% of the subjects had a relatively low individual income WTP for occupational safety and health.

After analyzing the subjects with different identities, we found that they all gave fairly little attention to occupational safety and health, where front-line mine staff who worked in the most difficult occupational environment and who had the greatest susceptibility to occupational diseases had the lowest level of attention to occupational safety and health (M = 2.331). More than 80% of the mine staff were not concerned about occupational safety and health, and this lack of concern needs to be addressed. Employees in third-party social institutions had relatively high levels of attention for occupational safety and health, particularly occupational diseases (M > 3). In terms of the WTP for occupational safety and health, employees in third-party social institutions had the highest WTP for an occupational safety and health tax WTP, whereas front-line mine staff have the lowest support degree. Government officials have the highest occupational safety and health individual income WTP, whereas the common public have the lowest occupational safety and health individual income WTP, with a mean value of 2.361.

### 5.3. WTP Analysis with Different Identities

**Figure 3:**
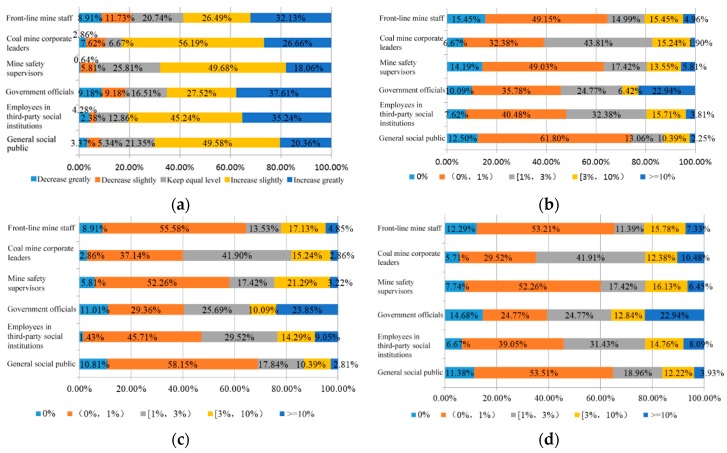
Occupation safety and health WTP of subjects with different identity. (**a**) Occupation safety and health tax WTP; (**b**) Occupation safety and health item WTP; (**c**) Occupation safety and health subject WTP; (**d**) Occupation safety and health event WTP.

The elaborate analysis of the WTP of subjects with a different identity was further explored, which is shown in Figure 3.

Throughout the further analysis on the WTP of subjects with a different identity, in comparison with the central financial expenditure budget for public safety (stability maintenance and emergency), about 60% of the subjects with different identities considered that central government should increase expenditure on occupational safety and health (including “increase slightly” and “increase greatly”), i.e., 82.85% of corporate mine leader subjects, 80.48% of employees in third-party social institutions, and 58.62% of front-line mine staff, who had a relatively high WTP for occupational safety and health measures. It should be noted that 37.61% of the government officials considered that it is necessary to greatly increase occupational safety and health funding, and the public safety financial expenditure level should be the top priority.

According to the subjects with different identities, the WTP by most subjects for occupational safety and health was less than the 3% income tax level, where 15.45% of the coal mine staff were willing to pay 0% to help the state solve occupational safety and health problems, which was the highest proportion, while 61.80% of the general public were willing to pay 0–1% (excluding 0% and 1% WTP) and 43.81% of coal mine corporate leaders were willing to pay 1–3% (excluding 3% WTP). It should be noted that that 22.94% of government officials were willing to pay 10% of their income for occupational safety and health. It is apparent that this proportion was higher than that of other subjects with a different identity.

After comparing the WTP for occupational safety and health, and the event WTP for subjects with different identities, we found that the WTP by subjects with different identities was similar for occupational disease patients and major safety accidents, where more than 50% of the general public, mine safety supervisors, and front-line mine staff were willing to pay less than 1% (excluding 0% and 1%), while approximately 40% of coal mine corporate leaders were willing to pay 1–3% for occupational disease patients and major safety accidents (excluding 3%), and more than 20% of government officials were willing to pay over 10%, which was the highest proportion among the six groups.

### 5.4. Occupational Safety and Health “Attention-WTP” Analysis

According to the questionnaire, we selected attention to occupational safety and health, tax WTP for occupational safety and health, and individual income WTP for occupational safety and health as targets for the statistical analysis. Over 3 points (excluding 3) denoted high attention, high tax WTP, and high individual income WTP, whereas less than 3 points (excluding 3) indicated low attention, low tax WTP, and low individual income WTP. With respect to the “attention-WTP” analysis, the researchers identified eight groups comprising “attention—government payment”, “no attention—government payment”, “attention—refusal of government payment”, “no attention—refusal of government payment”, “attention—individual payment”, “no attention—individual payment”, “attention—refusal of individual payment”, “no attention—refusal of individual payment”, all of which are explained as follows.

**Figure 4:**
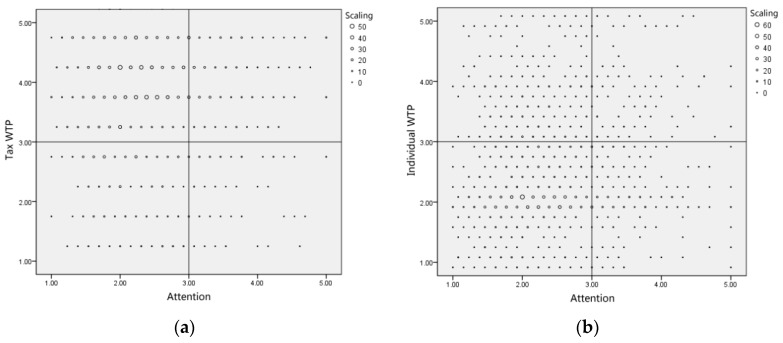
Statistical analysis of public attention and WTP to occupational safety and health. (**a**) Cross statistics of attention and tax WTP; (**b**) Cross statistics of attention and individual income WTP.

As shown in Figure 4a, only 13.48% of the subjects had double high characteristics in terms of attention to occupational safety and health and tax WTP, where they gave high attention to occupational safety and health, and they expected the government to pay. In addition, 10.94% of the subjects had double low characteristics in terms of attention to occupational safety and health and tax WTP, such that they paid a low level of attention to occupational safety and health, and believed that the government should not pay. Interestingly, although over 50% (50.83%) of participants paid a low level of attention to occupational safety and health, they expected the government to pay (not attention—government payment). In contrast, 2.40% of participants paid a high level of attention to occupational safety and health, though they did not expect the government to pay (attention—refusal of government payment).

As shown in Figure 4b, only 5.79% of the subjects had double-high characteristics, such that they paid a high level of attention to occupational safety and health, and were willing to contribute a portion of their individual income in payment (attention–individual payment). More than 50% (52.32%) of the subjects (with the highest proportion) had double-low characteristics, such that they paid a low level of attention to occupational safety and health and were not willing to pay from their individual income (no attention—refusal of individual payment). With respect to their views on occupational safety and health, it is interesting that just 15.80% of respondents could be classified and assigned to the “no attention—individual payment category”, which was far lower than the proportion of subjects who considered that occupational safety and health was a “not attention—government payment” (50.83%).

**Figure 5:**
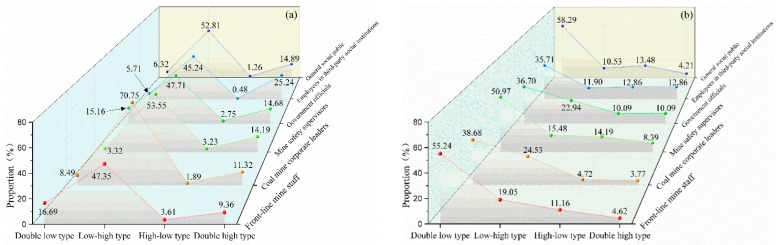
Analysis of attention to occupational safety and health and WTP by subjects with different identities. (**a**) Cross analysis of attention and tax WTP; (**b**) Cross analysis of attention and individual income WTP.

Next, we analyzed the attention to occupational safety and health and WTP for a tax, as well as the individual income WTP, where we obtained the following results (Figure 5).

As shown in Figure 5a, compared with the subjects with other identities, coal mine corporate leaders had the maximum proportion of low-high characteristics (70.75%), which means that they gave the minimum attention to occupational safety and health, but had the maximum WTP for a tax. Employees in third-party social institutions had the maximum double-high characteristics (25.24%), which means that they gave the greater attention and had the highest WTP for a tax to fund occupational safety and health. Front-line mine staff had the highest proportion of double-low characteristics (16.69%). The six subject types all had low proportions for high attention to occupational safety and health but a low WTP for a tax with high-low characteristics. 

As shown in Figure 5b, compared with the other identities, the general public had the maximum proportion with double-low characteristics (58.29%), which means that they gave little attention to occupational safety and health, and their individual income WTP was low. Coal mine corporate leaders had the maximum proportion of high-low characteristics (24.53%), which means that they gave little attention to occupational safety and health but had a high individual income WTP. Employees in third-party social institutions had the maximum proportion of double-high characteristics (12.86%), which means that they gave great attention to occupational safety and health, and their individual income WTP was high. Mine safety supervisors had the highest proportion of the high-low type (14.19%).

**Figure 6:**
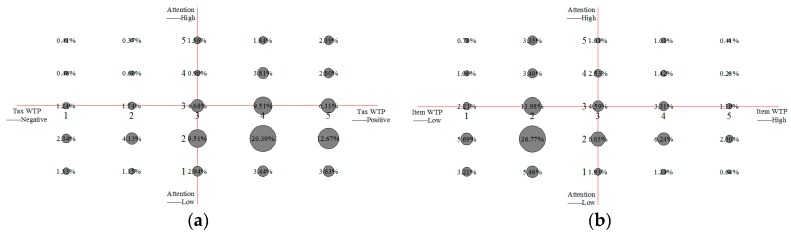
Analysis of attention and WTP to public occupational safety and health connotation. (**a**) Cross analysis of connotation attention and tax WTP; (**b**) Cross analysis of connotation attention and item WTP.

In order to understand the relationship between attention to occupational safety and health and WTP, we performed further analyses. However, due to limitations on length, we only present the analysis of attention to occupational safety and health connotation, tax WTP, and item WTP (the author can provide the other results on request). The specific analysis results will be presented as follows (Figure 6).

As shown in Figure 6a, 24.47% of the subjects had consistent characteristics in terms of attention to occupational safety and health connotation and tax WTP, and 40.13% of the subjects gave low attention to occupational safety and health connotation, but they had the highest tax WTP. 

As shown in Figure 6b, 48.79% of the subjects had consistent characteristics in terms of attention to occupational safety and health connotation and item WTP, and 41.13% of the subjects gave low attention to occupational safety and health connotation, but they had the highest item WTP. 

### 5.5. Correlation Analysis

We also explored the correlations between attention to occupational safety and health and item WTP, and the results are presented in Table 6.

There were significant positive correlations among attention to occupational safety and health connotation, individual income WTP, item WTP, subject WTP, and event WTP, among the subjects with different identities. Furthermore, there were significant positive correlations among attention to occupational safety and health connotation, individual income WTP, item WTP, subject WTP, and event WTP. There were also significant positive correlations among attention to occupational safety and health present condition, importance attention, system attention, occupational disease attention, individual income WTP, item WTP, subject WTP, and event WTP. 

### 5.6. Regression Analysis

Multiple linear regression was used to determine the predictions from the attention of occupational safety and health on the WTP with different identities. We treated identity as the control variable, and performed regression analysis based on dependent variables comprising the individual income WTP, item WTP, subject WTP, and event WTP, where the independent variable was attention. The results are shown in Table 7.

As shown in Table 7, the foregoing results demonstrated that attention to occupational safety and health was a significant predictor of WTP for occupational safety and health improvements. The coefficients for the effect of attention to occupational safety and health on the variable were: individual income WTP = 0.133 (*p* < 0.000), item WTP = 0.130 (*p* < 0.000), subject WTP = 0.121 (*p* < 0.000), and event WTP = 0.150 (*p* < 0.000).

## 6. Discussion

This study showed that the public generally give little attention to occupational safety and health, and individual income WTP for occupational safety and health improvements varied among subjects with different identities. In terms of attention, Chen et al. investigated the cognition status of teachers’ occupational safety knowledge in nursing, and found that 22.2% of teachers did not understand nursing occupational safety knowledge, and that 93.6% of students had been injured by sharp instruments during their practice in hospital [41]. Deng studied the attention to environmental health problems, and pointed out that college students’ attention to environmental problems should be improved, especially to the correct cognition of health hazards caused by environmental pollution [42]. This conclusion was highly consistent with the low public attention to occupational safety and health concluded in this research. In terms of WTP, few scholars have studied the WTP for occupational safety and health. In other public affair fields, Sun et al. found that 57.8% of the residents made voluntary contributions for environmental construction [30]. Yang et al. found that 55% of residents in Suzhou refused to pay for CO_2_ emission reduction, and their WTP increased with an increase of CO_2_ emission reduction ratios [43]. Soon and Ahmad studied residents’ WTP renewable energy and found that urban residents and North American households would have higher WTP, while Asian households have lower WTP [31]. These studies provided a reference for the study of public WTP for occupational safety and health.

This study showed that in coal mining companies, which have the highest rate of work-related accidents, the front-line mine staff gave the least attention to general occupational safety and health, including the current conditions, the importance of safety, and the system. Safety should be prioritized in coal mine because unsafe human behavior is the key cause of coal mine accidents in China [44]. Consequently, coal mine companies are expected to start from the beginning. They should enhance the front-line mine staff’s safety and health knowledge education, clarify present occupational safety and health conditions and importance, and reduce the unsafe behaviors of mine staff.

In terms of WTP, the public were willing to pay for occupational safety and health improvements via taxes. The public WTP was much lower when asked if they would be willing to pay directly for occupational safety and health improvements from their individual income. Our analysis of attention and WTP showed that more than 50% of the subjects regarded occupational safety and health as non-individual issues, but they supported the government in addressing occupational safety and health problems via taxes. Thus, although government financial revenues come mainly from taxes, funding for occupational safety and health was still considered indirect income expenditure, whereas individuals paying for occupational safety and health belongs to the category of direct income expenditure, with relatively smaller consumption pressure sensitivity. As the key explanatory factor influencing consumption behaviors, consumption pressure sensitivity is a hot research topic in the present consumption behavior research field. Pressure sensitivity has prominent influences on consumption behaviors because consumers will strategically distribute their assets in consumption behaviors under a pressure context [45,46]. Therefore, in order to address occupational safety and health problems, decision makers could consider adding occupational safety and health protection to the agenda for financial expenditure, and providing centralized governance.

Compared with the economic output, the occupational safety and health output is less obvious to the public, where improvements require common sense and collaboration among multiple public and private departments, collectives, and individuals. Mobilizing multiple groups to improve the governance of occupational safety and health problems is difficult according to previous studies. We found that there was a significant correlation between public attention to occupational safety and health and the individual income WTP for improvements. Previous studies have shown that the attention of investors to the stock market will directly influence their transaction behaviors [47]. Similarly, He showed that awareness of their illness among cancer patients will affect their psychological state [48]. In a study of socially responsible activities, Piamtechakun found that there were positive correlations among knowledge, understanding, attitudes, and participation behaviors during social responsible activities [49]. Thus, our study contributes to research into public attention and WTP research. In addition, resident cognition and support have major impacts on policy implementation. The present study provides insights into the governance of occupational safety and health, as well as enhancing public education and improving public attention to address occupational safety and health problems.

## 7. Conclusions

In general, the public are not concerned with occupational safety and health at present. Subjects with different identities also paid little attention to occupational safety and health. Employees in third-party social institutions gave the most attention to occupational safety and health, and they cared the most about occupational diseases, whereas the staff in mines showed the minimum level of attention to occupational safety and health. Over 80% of the staff in mines were not concerned about occupational safety and health, and this problem should be addressed.

In terms of the WTP for occupational safety and health improvements, the mean WTP value for members of the public was 3.746 and the individual income WTP was only 2.529. In addition: item WTP < subject WTP < event WTP. According to our analysis of subjects with different identities, employees in third-party social institutions had the maximum WTP for a tax for occupational safety and health (M = 4.048), where 80.48% of these subjects agreed that more of the central financial expenditure budget should be allocated to occupational safety and health (including slight increases and great increases). Front-line mine staff had the lowest WTP for a tax (M = 3.612) and less than 60% of them considered that more of the central financial expenditure budget should be allocated to occupational safety and health. Government officials had the maximum individual income WTP for occupational safety and health (M = 3.025), whereas the general public had the lowest (M = 2.361). Government officials had the maximum WTP for occupational safety and health (M = 2.963) and 22.94% of them were willing to pay 10% of their individual earnings to address occupational safety and health problems. The general public had the lowest item WTP for occupational safety and health (M = 2.281) and 61.80% were only willing to pay 0–1% for occupational safety and health (excluding 0% and 1%). Government officials had the maximum WTP for occupational safety and health subject WTP and event WTP, whereas the general public had the minimum WTP for occupational safety and health subject WTP and event WTP. Moreover, subjects with different identities were similar in terms of their WTP for occupational diseases and major safety accidents.

According to our analysis on occupational safety and health attention and WTP, 50.83% of the subjects considered occupational safety and health problems as a “not attention—government payment”, and only 15.80% of the subjects regarded occupational safety and health problems as a “not attention—individual payment”. There were 52.32% of subjects who regarded occupational safety and health problems as a “not attention—refusal of individual payment”. Further analysis proves that 24.47% of the subjects had consistent views regarding attention to occupational safety and health connotation and tax WTP, and 48.79% had consistent views of attention to occupational safety and health connotation, and item WTP.

Correlation analysis detected a significant correlation between occupational safety and health attention, individual income WTP, item WTP, subject WTP, and event WTP among subjects with a different identity.

Finally, the correlation analysis showed that attention to occupational safety and health was a significant predictor of occupational safety and health individual income WTP, item WTP, subject WTP, and event WTP, with coefficients of 0.133, 0.130, 0.121, and 0.150, respectively.

## Figures and Tables

**Table 1:** Occupational safety and health attention questionnaire example.

Dimensions	Items Descriptions	Degree of Attention				
Connotation attention	I pay attention to the issue of occupational safety and health and understand it to mean the conditions and factors affecting the health and safety of employees, temporary workers, visitors, and others in the workplace.	1	2	3	4	5
Present condition attention	I pay attention to the issue of occupational safety and health and believe that the current situation is not very positive.	1	2	3	4	5
Importance attention	I pay attention to the issue of occupational safety and health and believe that the health and safety of workers have become the core issue for China’s socialist modernization.	1	2	3	4	5
System attention	I pay attention to the issue of occupational safety and health and know that occupational health and safety management systems are designed to address occupational health and safety, rather than health and safety in areas such as employee fitness or health plans, product safety, loss of property or environmental impact.	1	2	3	4	5
Occupation illness attention	I pay attention to the issue of occupational safety and health and know that occupational diseases are diseases caused by exposure to dust, radioactive substances and other toxic and harmful substances in the occupational activities.	1	2	3	4	5

**Table 2:** Distribution of the WTP in terms of the bidding amounts regarding occupational safety and health WTP.

WTP (Income Proportion/Month/per Capita)	Sample Amount	Positive WTP Frequency (%)	Positive WTP Accumulation Frequency (%)	WTP Total Frequency (%)	Accumulation Frequency
0%	38			19.0	19.0
0.5%	11	6.8	6.8	5.5	24.5
1%	53	32.7	39.5	26.5	51.0
2%	10	6.2	45.7	5.0	56.0
3%	34	21.0	66.7	17.0	73.0
5%	18	11.1	77.8	9.0	82.0
10%	27	16.6	94.4	13.5	95.5
15%	3	1.9	96.3	1.5	97.0
20%	4	2.5	98.8	2.0	99.0
30%	2	1.2	100.0	1.0	100.0

**Table 3:** The structure of samples.

Demographic Variables		Percentage (%)	Demographic Variables		Percentage (%)
Gender	Male	74.69	Age	<20	0.34
	Female	25.31		21–30	28.12
Education	Primary school and following	2.56		31–40	32.48
	Junior middle school	16.89		41–50	23.30
	Senior high school	26.61		50–60	11.44
	Junior college	25.15		>60	4.32
	Bachelor degree	19.65	Marital status	Unmarried	12.82
	>Master degree	9.14		Married	82.91
Identity differences	Government officials	5.00		Divorced	2.89
	Coal mine corporate leaders	4.86		Bereaved	1.38
	mine safety supervisors	7.11	Politics status	Communists	27.66
	Front-line mine staff	40.71		Democratic parties	2.02
	Employees in third-party social institutions	9.64		Independent figure	7.08
	General public	32.68		Masses	63.24

**Table 4:** One-way ANOVA.

		A	CA	PCA	IA	SA	ODA	TWTP	IIWTP	IWTP	EWTP	SWTP
Identity	Sig.	**0.000**	**0.000**	**0.000**	**0.000**	**0.000**	**0.000**	**0.000**	**0.000**	**0.000**	**0.000**	**0.000**
Gender	Sig.	0.596	**0.000**	0.478	0.233	**0.009**	0.986	0.063	0.898	0.992	0.917	0.933
Age	Sig.	**0.020**	**0.005**	0.050	**0.011**	**0.001**	**0.006**	**0.009**	**0.000**	**0.000**	**0.000**	**0.041**
Marital status	Sig.	0.068	**0.019**	**0.005**	0.386	0.349	0.286	0.323	**0.023**	**0.014**	**0.037**	0.196
Monthly salary	Sig.	**0.000**	**0.000**	**0.000**	**0.000**	**0.000**	**0.000**	0.140	**0.000**	**0.000**	**0.000**	**0.003**
Residence area	Sig.	**0.000**	**0.002**	**0.000**	**0.000**	**0.000**	0.100	0.664	0.341	0.305	0.271	0.158
Education level	Sig.	**0.000**	**0.000**	**0.000**	**0.000**	**0.000**	**0.038**	0.158	**0.000**	**0.000**	**0.012**	**0.001**

Note: The bold font represents a significant level of *p* < 0.05. A: attention, CA: connotation attention, PCA: present condition attention, IA: importance attention, SA: system attention, ODA: occupation disease attention, TWTP: tax WTP, IIWTP: individual income WTP, IWTP: item WTP, EWTP: event WTP, SWTP: subject WTP.

**Table 5:** Occupational safety and health attention and WTP mean value distribution table of different subjects.

Variable	Front-Line Mine Staff		Coal Mine Corporate Leaders		Mine Safety Supervisors		Government Officials		Employees in Third-Party Social Institutions		General Social Public		Total	
	M	IV %	M	IV %	M	IV %	M	IV %	M	IV %	M	IV %	M	IV %
A	2.331	80.50	2.425	80.02	2.527	75.48	2.519	76.15	2.789	59.05	2.446	76.12	2.441	76.55
CA	2.550	59.19	2.698	46.23	2.819	42.58	2.119	80.73	2.791	49.52	2.239	71.49	2.478	61.50
PCA	2.229	80.95	2.230	78.30	2.414	76.13	2.373	86.24	2.662	60.48	2.349	77.25	2.330	77.60
IA	2.472	65.39	2.486	70.75	2.652	58.71	2.762	54.13	2.919	44.76	2.636	58.43	2.597	60.35
SA	2.078	79.26	2.392	70.75	2.368	70.32	2.725	55.96	2.626	50.48	2.468	63.90	2.325	69.25
ODA	2.636	59.86	2.849	37.74	2.755	56.13	2.706	55.05	3.202	34.76	2.629	58.99	2.710	55.58
TWTP	3.612	20.63	3.962	10.78	3.787	6.45	3.752	18.35	4.048	6.67	3.782	8.71	3.746	13.77
IIWTP	2.505	67.98	2.813	43.40	2.576	66.45	3.025	46.79	2.767	52.38	2.361	74.86	2.529	66.36
IWTP	2.453	64.60	2.733	38.68	2.477	63.23	2.963	45.87	2.676	48.10	2.281	74.30	2.459	63.88
SWTP	2.534	64.49	2.781	39.62	2.639	58.06	3.064	40.37	2.838	47.14	2.362	68.96	2.553	61.40
EWTP	2.526	65.50	2.924	34.91	2.613	60.00	3.046	39.45	2.786	45.71	2.438	64.89	2.574	60.21

Note: IV: inferior value, A: attention, CA: connotation attention, PCA: present condition attention, IA: importance attention, SA: system attention, ODA: occupation disease attention, TWTP: tax WTP, IIWTP: individual income WTP, IWTP: item WTP, EWTP: event WTP, SWTP: subject WTP.

**Table 6:** Correlations between attention to occupational safety and health connotation and WTP (n = 2179).

		TWTP	IIWTP	IWTP	SWTP	EWTP
A	Pearson Correlation	0.037	0.132 **	0.121**	0.108 **	0.121 **
	Sig. (2-tailed)	0.080	0.000	0.000	0.000	0.000
CA	Pearson Correlation	0.61 **	0.108 **	0.075 **	0.097 **	0.113 **
	Sig. (2-tailed)	0.005	0.000	0.000	0.000	0.000
PCA	Pearson Correlation	0.026	0.108 **	0.100 **	0.082 **	0.102 **
	Sig. (2-tailed)	0.225	0.000	0.000	0.000	0.000
IA	Pearson Correlation	0.035	0.133 **	0.110 **	0.083 **	0.106 **
	Sig. (2-tailed)	0.099	0.000	0.000	0.000	0.000
SA	Pearson Correlation	0.025	0.075 **	0.087 **	0.061 **	0.051 *
	Sig. (2-tailed)	0.239	0.000	0.000	0.004	0.016
ODA	Pearson Correlation	0.033	0.155 **	0.128 **	0.143 **	0.139 **
	Sig. (2-tailed)	0.120	0.000	0.000	0.000	0.000

Note: ** Significant correlation at *p* < 0.01 level, * significant correlation at *p* < 0.05 level. A: attention, CA: connotation attention, PCA: present condition attention, IA: importance attention, SA: system attention, ODA: occupation disease attention, TWTP: tax WTP, IIWTP: individual income WTP, IWTP: item WTP, EWTP: event WTP, SWTP: subject WTP.

**Table 7:** Regression between attention to occupational safety and health connotation and WTP (n = 2179).

	Individual Income WTP		Item WTP		Subject WTP		Event WTP	
	Model 1	Model 2	Model 3	Model 4	Model 5	Model 6	Model 7	Model 8
Identity discrepancy	0.068 ***	0.060 **	0.075 ***	0.068 **	0.065 **	0.058 **	0.064 **	0.055 **
Gender	0.046	0.038	0.048	0.041	0.053	0.046	0.036	0.028
Age	−0.046 ***	−0.046 ***	−0.052 ***	−0.053 ***	−0.049 **	−0.050 ***	−0.036 *	−0.036 *
Marital status	−0.022	−0.017	−0.040	−0.036	−0.002	0.001	−0.023	−0.018
Monthly salary	0.104 ***	0.092 ***	0.119 ***	0.106 ***	0.122 ***	0.111 ***	0.072 *	0.058 *
Residence area	−0.020	−0.025	−0.013	−0.018	−0.062 *	−0.066 *	0.016	0.011
Education level	0.053 **	0.043 *	0.084 ***	0.074 **	0.028	0.019	0.047	0.035
Attention		0.133 ***		0.130 ***		0.121 ***		0.150 ***
F	8.993	11.346	10.895	12.181	7.720	9.075	4.342	6.871
R^2^	0.028 ***	0.040 ***	0.034 ***	0.043 ***	0.024 ***	0.032 ***	0.014 ***	0.025 ***
△F	8.993	27.059	10.895	20.493	7.720	18.138	4.342	24.245
△R^2^	0.028 ***	0.012 ***	0.034 ***	0.009 ***	0.024 ***	0.008 ***	0.014 ***	0.011 ***

Note: *** *p* < 0.001, ** *p* < 0.01, * *p* < 0.05.

## Nomenclature

RMSEA	Root mean square error of approximation
GFI	Goodness-of-fit index
IFI	Incremental fit index
CFI	Comparative fit index
TLI	Tacker-Lewis index
ANOVA	Analysis of Variance

## Appendix A

**Table A1:** The 13 items for attention and the four items for WTP.

Dimensions	Items Descriptions
Connotation attention	V1. I pay attention to the issue of occupational safety and health and understand it to mean the conditions and factors affecting the health and safety of employees, temporary workers, visitors, and others in the workplace.
Present condition attention	V2. I pay attention to the issue of occupational safety and health and believe that the current situation is not very positive.
	V3. I pay attention to the issue of occupational safety and health and know that every 15 s, about 160 workers experience occupational accidents throughout the world and one worker will die due to occupational accidents or diseases.
	V4. I pay attention to the issue of occupational safety and health and know that over 16 million companies were engaged in poisonous and hazardous operations in 2011, and 200 million workers were affected by occupational diseases to varying degrees in the labor process.
	V5. I pay attention to the issue of occupational safety and health and know that industrial injury accidents and occupational diseases in China have reached to more than 200 billion RMB and account for 2.5% in GDP terms.
	V6. I pay attention to the issue of occupational safety and health and know that the occupational safety and health level in China lags behind the economic and social development.
	V7. I pay attention to the issue of occupational safety and health and know that the non-synchronized development exists between occupational safety and health governance, and specifically health governance lags behind safety governance in China.
Importance attention	V8. I pay attention to the issue of occupational safety and health and believe that the health and safety of workers have become the core issue for China’s modernization.
	V9. I pay attention to the issue of occupational safety and health and know that the people’s health should be placed on the priority of national development which was put forward in the National Hygiene and Health Conference held in 2016.
System attention	V10. I pay attention to the issue of occupational safety and health and know that occupational health and safety management system (OHSMS) is a modern mode of production safety management emerging in the world in the late 1980s, which is jointly called “the management method of the post-industrialization era” together with ISO 9000 and ISO 14000 standard systems.
	V11. I pay attention to the issue of occupational safety and health and know that occupational health and safety management systems are designed to address occupational health and safety, rather than health and safety in areas such as employee fitness or health plans, product safety, loss of property or environmental impact.
Occupation illness attention	V12. I pay attention to the issue of occupational safety and health and know that occupational diseases are diseases caused by exposure to dust, radioactive substances and other toxic and harmful substances in the occupational activities.
	V13. I pay attention to the issue of occupational safety and health and know that there are 26,000 new cases of occupational diseases in China, of which pneumoconiosis occupying 80% ranks the first place.
Tax WTP	The financial expenditure budget by the central government for public safety (stability maintenance and emergencies) in 2017 is 183.855 billion RMB and the corresponding per capita budget is 133.71 RMB. If the country added occupational safety and health to the agenda of the financial expenditure budget, should the per capita public safety level be increased or reduced in your opinion.
Item WTP	If you were required to help country solve occupational safety and health problems with your personal income (such as setting up special occupational safety and health funds and purchasing insurance for occupational disease patients), what percentage of your income would you be willing to pay?
Subject WTP	If there was a donation campaign for patients with occupational diseases (i.e., pneumoconiosis), what percentage of your income would you donate to these patients with occupational diseases?
Event WTP	If there happened a major safety accident somewhere (i.e., Tianjin Major Fire and Explosion Accident), and a donation was needed for post-disaster reconstruction work caused by the accident, what percentage of your income would you donate to support post-disaster reconstruction work?

## References

[1] Schulte P.A., Wagner G.R., Ostry A., Blanciforti L.A., Cutlip R.G., Krajnak K.M., Luster M., Munson A.E., O’Callaghan J.P., Park C.G. (2007). Work, obesity, and occupational safety and health. Am. J. Public Health.

[2] Zytoon M.A., Basahel A.M. (2017). Occupational safety and health conditions aboard small- and medium-size fishing vessels: Differences among age groups. Int. J. Environ. Res. Public Health.

[3] Hazavehei S., Shadzi S., Asgari T., Pourabdian S., Hasanzadeh A. (2008). The effect of safety education based on health belief model (HBM) on the workers practice of borujen industrial town in using the personal protection respiratory equipments. PLoS ONE.

[4] (2016). State Administration of Work Safety (EB/OL). http://www.chinasafety.gov.cn/newpage/Contents/Channel_20132/2012/0428/169760/content_169760.htm.

[5] Tajfel H. (1972). International Encyclopedia of the Social & Behavioral Sciences. Soc. Categoriz..

[6] Guo Y.J. (2002). Identity system and the concept structure of Chinese people. Philos. Trends.

[7] Kowalski T.R.K.M., Vaught C., Mcwilliams L.J., Reissman D.B., Burke R.J., Clarke S. (2011). Psychological and behavioral aspects of occupational safety and health in the US coal mining industry. Occupational Health and Safety.

[8] Zhao W., Liu T. (2015). Promoting dustless coal mine construction to strengthen the management of occupational safety and health. Shandong Coal Sci. Technol..

[9] Bundestag D. (2007). Report of the federal government on occupational safety and health and on occupational accident and disease occurrences in the federal republic of Germany in the year 2006. Biomacromolecules.

[10] Ratkiewicz J., Menczer F., Fortunato S., Flammini A., Vespignani A. Traffic in Social Media II: Modeling Bursty Popularity. Proceedings of the IEEE Second International Conference on Social Computing.

[11] Spijkers O., Honniball A. (2015). Developing global public participation. Int. Community Law Rev..

[12] Krishna A. (1991). Effect of dealing patterns on consumer perceptions of deal frequency and willingness to pay. J. Mark. Res..

[13] Wu L., Wang H., Zhu D., Hu W., Wang S. (2016). Chinese consumers’ willingness to pay for pork traceability information—The case of Wuxi. Agric. Econ..

[14] Janssen M., Hamm U. (2012). Product labelling in the market for organic food: Consumer preferences and willingness-to-pay for different organic certification logos. Food Qual. Preference.

[15] Benjamin S.C.U., Obinna E.O., Nkoli P.U., Maduka D.U., Ogochukwu P.E. (2010). Willingness to pay for rapid diagnostic tests for the diagnosis and treatment of malaria in southeast Nigeria: Ex post and ex ante. Int. J. Equity Health.

[16] Davvetas V., Sichtmann C., Diamantopoulos A. (2015). The impact of perceived brand globalness on consumers’ willingness to pay. Int. J. Res. Mark..

[17] Wang K., Wu J., Wang R., Yang Y., Chen R., Maddock J.E., Lu Y. (2015). Analysis of residents’ willingness to pay to reduce air pollution to improve children’s health in community and hospital settings in Shanghai, China. Sci. Total Environ..

[18] Hackbarth A., Madlener R. (2016). Willingness-to-pay for alternative fuel vehicle characteristics: A stated choice study for Germany. Transp. Res. Part A.

[19] Sundt S., Rehdanz K. (2015). Consumers’ willingness to pay for green electricity: A meta-analysis of the literature. Energy Econ..

[20] Beltramo T., Blalock G., Levine D.I., Simons A.M. (2015). The effect of marketing messages and payment over time on willingness to pay for fuel-efficient cookstoves. J. Econ. Behav. Organ..

[21] Shultz S.D., Lindsay B.E. (2015). The willingness to pay for groundwater protection. Water Resour. Res..

[22] Gunatilake H., Yang J.C., Pattanayak S., van den Berg C. (2015). Willingness-to-Pay and Design of Water Supply and Sanitation Projects: A Case Study.

[23] Vringer K., Aalbers T., Blok K. (2007). Household energy requirement and value patterns. Energy Policy.

[24] Groot J.D., Steg L. (2007). General beliefs and the theory of planned behavior: The role of environmental concerns in the TPB. J. Appl. Soc. Psychol..

[25] Seasholes M.S., Wu G. (2007). Predictable behavior, profits, and attention. J. Empir. Financ..

[26] Shao Y. (2015). Research on Rhetorical Strategies and Communication Paths of News Broadcast under the Perception of Public. Ph.D. Thesis.

[27] (2016). National Bureau of Statistics of China [EB/OL]. http://data.stats.gov.cn/easyquery.htm?cn=C01&zb=A0403&sj=2015.

[28] Li C.M., Liu G.H. (2013). Factor Analysis on Public Participation Awareness—A Sample from Chengdu. J. Southwest Jiaotong Univ..

[29] Li L. (2013). The study on the influencing mechanism of films and TV dramas on the travel intention of urban residents—Taking films and TV dramas shot in Qingdao as an example. J. Qingdao Tech. Coll..

[30] Sun Y., Wang W., Fang S., Wang Y. (2014). An empirical analysis on influencing factors of residents’ environment willingness to pay under the background of the plains afforestation project—Taking Beijing as an example. For. Econ..

[31] Soon J.J., Ahmad S.A. (2015). Willingly or grudgingly? A meta-analysis on the willingness-to-pay for renewable energy use. Renew. Sustain. Energy Rev..

[32] Breidert C., Hahsler M., Reutterer T. (2006). A review of methods to measuring Willingness-to-pay. Innov. Mark..

[33] Tao Z., Yan H., Zhan J. (2012). Economic valuation of forest ecosystem services in heshui watershed using contingent valuation method. Procedia Environ. Sci..

[34] Lv J.-J., Xu A.-Q., Wang J. (2011). A review of the application of contingent valuation method in public health research. Chin. J. Health Policy.

[35] Wang H.M., Ni C.J., Xu R.Z. (2011). On Consumer’s Willingness to Pay on Food Quality and Label Safety: A Case Study of Pork Consumption in Nanjing City. J. Nanjing Agric. Univ..

[36] Mee-Ok K., Kun-Sei L., Jung-Hoe K., Ji-Soo J. (2011). Willingness to pay for hospice care using the contingent valuation method. Yonsei Med. J..

[37] Flick U. (2009). An Introduction to Qualitative Research.

[38] Chen F., Chen H., Guo D., Long R. (2017). Analysis of undesired environmental behavior among Chinese undergraduates. J. Clean. Prod..

[39] O’Brien B., Gafni A. (1996). When do the “dollars” make sense? Toward a conceptual framework for contingent valuation studies in health care. Med. Decis. Mak..

[40] Boccaletti S., Nardella M. (2000). Consumer willingness to pay for pesticide-free fresh fruit and vegetables in Italy. Int. Food Agribus. Manag. Rev..

[41] Chen L.H., Ren S., Zhang Y.H. (2010). Investigation on the status quo of teachers’ knowledge of nursing occupational safety in secondary health schools. Pract. Prev. Med..

[42] Deng F.R., Wang X., Lu X.L., Guo X.B. (2011). Investigation and Analysis on Knowledge, Attitude and Practice of Environmental Health among Undergraduate Students in Beijing. J. Environ. Health.

[43] Yang N. (2012). The Application of Foreing Curriculum in Higher Vocational College. Ph.D. Thesis.

[44] Chen H. (2006). Study on Unsafe Behavior in Major Coal Mine Accidents in China.

[45] Durante K.M., Laran J. (2016). The effect of stress on consumer saving and spending. J. Mark. Res..

[46] Tian L., Liu Z.Y. (2017). Can Endowment Insurance Effectively Relieve Residents’ Perception of Consumption Stress?—Empirical Evidence Based on the CGSS. China Soft Sci..

[47] Zhi D.A., Engelberg J., Gao P. (2011). In search of attention. J. Financ..

[48] He C.Y. (2016). The influence of the degree of knowledge on the mental state of the patients with gastric cancer and the nursing intervention. Chin. J. Clin..

[49] Piamtechakun R. (2017). Public relations media of the crown property bureau towards attitude and behavior in social responsibility participation willingness to pay. Int. J. Res. Mark..

